# Meta-Analysis of ERCC1 Protein Expression and Platinum Chemosensitivity in Non-Small-Cell Lung Cancer

**DOI:** 10.1155/2020/7376568

**Published:** 2020-04-30

**Authors:** Guoping Li, Dan Cheng

**Affiliations:** ^1^Department of Respiration, Tongde Hospital of Zhejiang Province, No. 234 Gucui Road, Xihu District, Hangzhou, Zhejiang 310012, China; ^2^Department of Respiration, Central Hospital of Haining, Zhejiang Province People's Hospital Haining Hospital, No. 758 Chang'an Road, Chang'an Town, Haining, Zhejiang 314408, China

## Abstract

**Objective:**

To carry out the meta-analysis on the relationship between the expression of nucleotide excision repair cross-complementary enzyme 1 (ERCC1) protein and platinum chemosensitivity in patients with advanced non-small-cell lung cancer (NSCLC).

**Methods:**

The literature on the expression of ERCC1 and platinum chemosensitivity in patients with advanced NSCLC was searched in computer, which was published from January 2009 to August 2019 on the databases such as China Journal Full-text Database (CJFD), China National Knowledge Infrastructure (CNKI), Wanfang Database, VIP, PubMed, EMBASE, and others. Stata 15.0 was used for statistical analysis, and ethnicity subgroup analysis was taken.

**Results:**

Finally, 14 studies were included and 1337 patients were involved, of which 697 were ERCC1 positive, with a positive rate of 53.5%. The combined OR was 0.53 (95% CI: 0.30∼0.79; *P* < 0.01). The results of ethnicity subgroup analysis showed that there was no significant difference, with OR of 0.50 (95% CI: 0.31∼0.82; *P*=0.001) in Asian population and OR of 0.56 (95% CI: 0.30∼1.07) in Caucasian population.

**Conclusion:**

The sensitivity to platinum chemotherapy in patients with ERCC1 protein negative expression in the middle and late stages of NSCLC is better than that in patients with positive expression, especially in Asian population. There is no correlation in Caucasian population.

## 1. Introduction

Lung cancer is one of the most common malignant tumors at present, and the number of patients with which is increasing rapidly all over the world. It has the highest mortality rate in the world, with the annual deaths of more than 1 million [1]. In addition, according to some relevant reports, it is currently the fastest growing disease in the world in terms of morbidity and mortality, posing a serious threat to human health. According to the pathological features, the lung cancer can be divided into small-cell lung carcinoma (SCLC) and non-small-cell lung carcinoma (NSCLC) [2, 3], among which NSCLC is the major one, mainly including squamous cell carcinoma, adenocarcinoma, and large cell carcinoma. In the past 20 years, the morbidity of lung cancer in China's rural regions as well as the cities is increasing significantly, especially that of NSCLC [4, 5]. Nowadays, the clinical treatment of lung cancer is based on platinum drugs. There is no significant difference in the therapeutic effect of these platinum-containing regimens. The drug response rate is only 30% to 40%, and the median survival time is 8–10 months; the 5-year survival rate is less than 15% [6]. Clinical studies have shown that the prognosis of advanced NSCLC chemotherapy is related to stage, sex, age, physical status (PS), and other factors, but these clinical factors are obviously not accurate enough. The sensitivity of NSCLC with the same pathological type and stage to the same chemotherapy is significantly different. With the advance of pharmacogenetics and pharmacogenomics, we can study on the level of single-nucleotide gene polymorphism of patient gene and gene expression of tumor tissue, so that the more accurate prediction of sensitivity and prognosis of chemotherapy in patients can be obtained [7, 8]. As the theoretical knowledge is accumulated and improved, the focus of clinical research of related scholars will be to develop a treatment scheme that can further improve the pertinence of platinum drugs on top of the existing treatment level. Tumor is resulting from abnormal mechanism of cell differentiation, proliferation, and death. Although the mechanism of tumorigenesis is not so clear, it is known that the damage of DNA, the abnormality of gene structure, and the resulting changes in the expression or function of oncogenes as well as tumor suppressor genes are the necessary prerequisites for malignant transformation of cells. Therefore, DNA repair is a protective response of living cells to a variety of damage to their genomes through various pathways. To a large extent, it ensures the stability, replication, and transcription fidelity along with the accuracy of genetic material and makes the life activities proceeding normally. However, many anticancer cytotoxic drugs target cell DNA, so the abnormal repair ability of it is closely related to the formation of tumor resistance [9]. The development of tumor cells to platinum resistance is caused by many factors, which may include the decrease of drug accumulation, the increase of glutathione level as well as metallothionein, and the improvement of DNA repair capacity (DRC) [10]. Among them, DRC is a very important influencing factor. The repair capacity of damaged DNA has great variability among different individuals and at different ages, which leads to the difference of sensitivity of tumor cells to DNA-related cytotoxic drugs [11]. In recent years, basic studies have indicated that nucleotide excision repair system (NER) is one of the main repair pathways after platinum-induced DNA damage in tumor cells [12]. Excision repair cross-complementation group 1 (ERCC1), a destructive repair enzyme of DNA, is a key factor in NER, and its expression is an important factor in the sensitivity of platinum drugs [13]. At present, there are many academic studies on the relationship between ERCC1 protein expression and platinum chemosensitivity of lung cancer, but the results are inconsistent or controversial. Therefore, in this study, meta-analysis was used to explore their relationship in patients with advanced NSCLC in order to avoid blind selection of chemotherapy regimens and provide a theoretical basis for clinical treatment.

## 2. Material and Methods

### 2.1. Literature Retrieval

With the keywords of lung cancer, ERCC1, and platinum, the literature from January 2009 to August 2019 was searched in the databases such as CJFD, CNKI, Wanfang Database, VIP, PubMed, and EMBASE. The preliminary screening was done mainly by reading the abstracts. And then the references of the literature that met the inclusion criteria were carefully read to get the wanted articles. The selected literature was published in Chinese or English.

### 2.2. Selection Criteria

#### 2.2.1. Literature Inclusion Criteria

(1) The subjects were patients with advanced NSCLC, that is, patients at clinical stages II–IV. (2) The case-control study on the expression of ERCC1 protein and efficacy of NSCLC in platinum chemotherapy was analyzed. (3) NSCLC was primary lung cancer and diagnosed by pathology or cytology. (4) The test method of the protein expression of ERCC1 was immunohistochemical (IHC) staining. (5) The WHO standard was used as the evaluation standard of curative effect, with the complete remission + partial mitigation as the total effective rate. (6) For the multiple reports of the same author, the latest or most complete reports were used. (7) The Chinese literature was those included in the Core Database of Peking University Library.

#### 2.2.2. Literature Exclusion Criteria

(1) The study was on animal experiment or the lung cancer cell line. (2) The study was on the nonprimary lung cancer, such as metastatic or recurrent cancer. (3) The study was on the SCLC. (4) The study was about chemotherapy with nonplatinum regimen, with less than 2 courses of chemotherapy. (5) The study was on the relationship between the expression of ERCC1 protein and the efficacy of platinum chemotherapy, such as ERCC1 mRNA and ERCC1 gene polymorphism.

### 2.3. Data Extraction and Quality Evaluation

All the following information was extracted from the included literature: (1) general information: it includes researchers, publication time, and the country; (2) the literature characteristics: clinical staging, chemotherapy regimen, the number of ERCC1-positive and ERCC1-negative cases, and the number of effective cases of chemotherapy; and (3) methodological features: it includes ERCC1 detecting methods, cutoff value, statistical methods, and results. As the inclusion of the literature is retrospective studies, there are no random and blind requirements. The quality of the literature was evaluated according to the Newcastle–Ottawa Scale (NOS) [14], with low-quality ones under 6 stars and high-quality ones of 6 stars or above. Only the literature of high quality was included in this study. Two evaluators evaluated the literature independently and cross-checked. When there were divergences, the consensus was reached through discussion.

### 2.4. Statistical Methods

The meta-analysis was carried out by Stata 15.0 software. The OR and 95% CI, as the effect size, were calculated to represent the results. *Q* test was used to test the heterogeneity of the results. If *I*^2^ ≥ 50% or *P* ≤ 0.05, it is considered that there was heterogeneity, and the random-effects model was used. If *I*^2^ < 50% and *P* > 0.05, it was considered that there was no heterogeneity, and the fixed-effects model could be used for data consolidation. *Z* test was used to test the significance of the combined OR value. In this meta-analysis, the evaluation of publication bias of the included literature was judged by whether the funnel plot was symmetrical or not. Funnel plot was to use the standard error of each study log (OR) to draw its OR value. If the funnel plot was asymmetric, there may be publication bias. Egger's test and Begg's test were used to test the publication bias.

## 3. Results

### 3.1. Literature Retrieval Results

**Figure 1:**
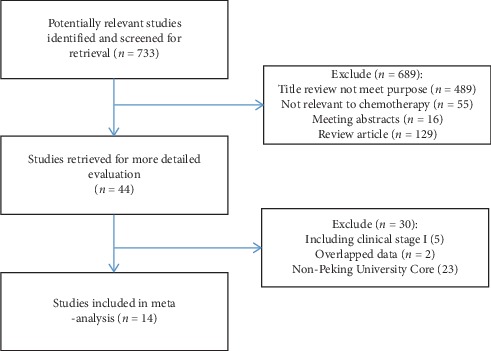
A flow diagram of the study selection process.

The databases were searched comprehensively, and then, the retrieval results were cross-checked. The selected literature was screened in strict accordance with the exclusion criteria and the quality control requirements after reading carefully. At the end, 14 articles [15–28] were chosen into the meta-analysis, with the specific screening process in Figure 1 and the basic characteristics of the included literature in Table 1. Of the 1337 patients with non-small-cell lung cancer, 697 were positive, with a positive expression rate of 53.5%.

### 3.2. Relationship between ERCC1 Expression and Sensitivity to Platinum Chemotherapy in NSCLC

**Figure 2:**
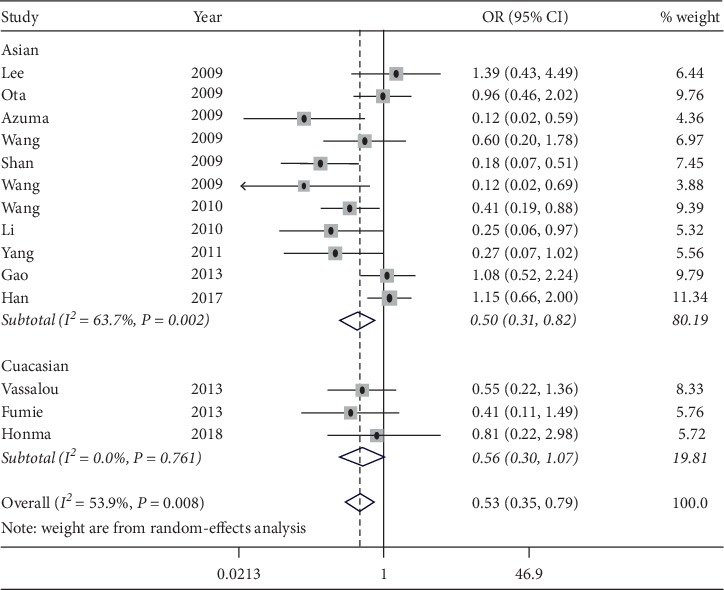
Forest plot for the association between ERCC1 expression and platinum chemosensitivity of NSCLC.

The meta-analysis of the included 14 studies showed that there was statistical heterogeneity among the studies (*P* ≤ 0.001; *I*^2^ = 61.2%). Using the random-effect model to analysis, the forest plot was obtained, which is shown in Figure 2. The complete remission + partial remission rate of 14 studies (1337 cases) reported in patients with NSCLC after chemotherapy indicated that the patients with high expression of ERCC1 were less sensitive to chemotherapy than those with low expression, with the combined OR of 0.53 (95% CI: 0.30∼0.79; *P* < 0.01). The results of ethnicity subgroup analysis showed that, for Asian population, there was a significant difference with the combined OR of 0.50 (95% CI: 0.31∼0.82; *P*=0.001). For Caucasian population, with the combined OR of 0.56 (95% CI: 0.30∼1.07), the difference was not statistically significant, as shown in Table 2. The results demonstrated that there was a correlation between the expression of ERCC1 and platinum sensitivity in patients with advanced NSCLC. The chemotherapy sensitivity of patients with low expression of ERCC1 was higher than that of patients with high expression of platinum, especially in Asian population. However, no correlation was found in the Caucasian population. In order to better explore the source of heterogeneity, we conducted a subgroup analysis of tumor staging, excluding patients with stage II of non-small-cell lung cancer, and then analyzed. The results showed that (Table 2), stage III-IV patients were analyzed alone, and heterogeneity decreased, OR = 0.57 (95% CI: 0.39–0.84, *P* < 0.05). This showed that tumor staging was one of the sources of heterogeneity.

### 3.3. Publication Bias Analysis

**Figure 3:**
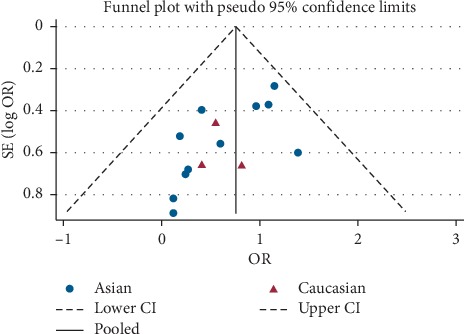
Funnel plot for the assessment of publication bias.

The literature was analyzed by funnel plot, and the symmetry of the plot was analyzed by Egger's test and Begg's test. The specific results are shown in Table 2 and Figure 3, which indicated that the funnel plot was basically symmetrical, and the *P* value of Begg's test was more than 0.05, while the *P* value of Egger's test was slightly less than 0.05 so that there was partial publication bias.

### 3.4. Sensitivity Analysis

**Figure 4:**
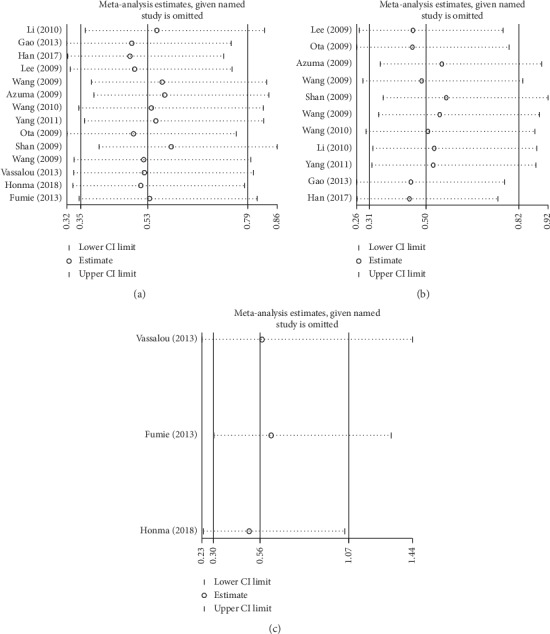
Sensitivity analysis results. (a). Overall. (b). Asian. (c). Caucasian.

The results of sensitivity analysis are shown in Figure 4. Each study was excluded one by one, and the results of meta-analysis showed that there was no significant change in the combined effect. The results of sensitivity analysis were also stable in Asian population and Caucasian population, meaning that the included 14 articles were stable.

## 4. Discussions

Platinum resistance is caused by a variety of factors, including the decrease of drug accumulation, the increase of drug detoxification (such as glutathione and metallothionein), the enhancement of DRC, and the increase of platinum-DNA adducts [29]. Besides, a number of studies have shown that DNA repair is the main cause of platinum resistance in the treatment of platinum drugs. DRC is the capacity to stabilize the related cellular response by restoring the structure of normal DNA sequence and maintaining genetic information. DNA damage repair genes can repair DNA damage caused by different reasons, thus protecting the integrity of genetic information. In the process of cancer treatment, DNA is the target molecule of many kinds of anticancer drugs; therefore, its abnormal damage as well as repair ability is closely related to the formation of tumor drug resistance [30]. NER is the main way of DNA repair in mammalian cells, and it is a necessary factor to protect the host from tumor invasion, the low expression of which is associated with the susceptibility to many kinds of tumors [31]. Further research studies indicate that the antitumor effect of platinum drugs is not related to platinum-DNA interchain crosslink, but more closely to platinum-DNA chain, which is mainly repaired by NER system [32]. Platinum resistance is caused by the repair ability of DNA. The removal of platinum drugs through NER leading to DNA intrachain adducts is considered to be the main mechanism of platinum drug resistance [12]. Therefore, NER plays a key role in platinum resistance. Enhancement of NER is an important mechanism leading to cisplatin resistance [33]. ERCC1, a destructive repair enzyme of DNA, is one of the key enzymes in the main part of the helix complex, which is involved in the process of identifying the damaged site. ERCC1-XPD heterodimer formed by ERCC1 protein and XPD is a 5′-3′ DNA restriction endonuclease in NER [34]. In addition, it also plays a certain role in DNA repair connection and internal chain cross repair [35]. Some researchers have found that cisplatin-based chemotherapy can prolong the survival time of patients with no or relatively low expression of ERCC1 in NSCLC cells. In 2006, Olaussen [36] first reported that among the patients with NSCLC complete resection, those with low expression of ERCC1 in tumor specimens had a poor prognosis, but could benefit from chemotherapy containing cisplatin. Those with high expression of ERCC1 had a better prognosis, but it was difficult to benefit from adjuvant chemotherapy. After that, it has been confirmed that, in the early stage of NSCLC, the 5-year survival time and median survival time in the ERCC1 positive group are longer than those in the opposite group. It is considered that the low expression of ERCC1 is a sign of high invasiveness of tumor, and the prognosis is often poor [37]. However, the research of Sad et al. [38] showed that, in the late stage of NSCLC, the 5-year survival time and median survival time in the ERCC1 negative group were longer than those in the opposite group. By analyzing the relationship between the expression of ERCC1 protein in tumor resection samples of NSCLC patients at stage I∼IIIA and postoperative survival, and taking the postoperative survival time as the final index to study the difference of curative effect in the chemotherapy group, Wang et al. [39] discussed the significance of ERCC1 expression in judging the prognosis of different patients and the existence of cisplatin resistance. It was suggested that the prognosis of patients with ERCC1 (+) expression at stage I was better than that of patients with ERCC1 (–). On the contrary, for NSCLC patients with platinum chemotherapy in stage I and IIIA, the survival time of those with low ERCC1 expression was longer than that of patients with high ERCC1 expression. Thus, there may be biphasic reactions in different stages of NSCLC patients. In this way, the study conducts a meta-analysis on the relationship of the expression of ERCC1 and the chemosensitivity of platinum drugs in patients with advanced NSCLC, in order to comprehensively evaluate the comprehensive effect of ERCC1 protein expression on middle- and late-stage patients with NSCLC. Another purpose of the study is to provide evidence-based theory for clinical treatment.

The meta-analysis includes 14 articles [15–28], consisting of 1337 NSCLC patients, with the total positive rate of ERCC1 of 52.1%. The results show that there is a correlation between the high expression of ERCC1 and the chemosensitivity of platinum in the middle and late stages of NSCLC. The low expression of ERCC1 is more sensitive to platinum chemotherapy in the middle and late stages of NSCLC than that of high expression. The same results are found in Asian and Caucasian subgroups. From the perspective of statistical heterogeneity, the difference is statistically significant, so that there is a certain degree of statistical heterogeneity. In terms of publication bias, the funnel plot is basically symmetrical and Egger's test shows that the *P* value is slightly less than 0.05, so there is a partial publication bias. Each study is excluded one by one, and meta-analysis is used. Then, the sensitivity analysis shows that there is no significant change in the combined effect, indicating that the stability of the included study is good. It can be seen that the conclusion drawn from the study is relatively reliable. However, considering that there are few studies on Caucasian population, and their OR values are low, with the upper limit of 95% confidence interval only slightly greater than 1. Therefore, the conclusion of Caucasian population still needs to be treated with caution. The study of Dong et al. [40] on the expression of ERCC1 and the prognosis of platinum chemotherapy in patients with advanced NSCLC indicates that the 5-year survival rate and median survival time of patients with low expression of ERCC1 are higher than those of patients with high expression, which is consistent with the results of chemosensitivity in this study.

Of course, this study also has some limitations: (1) The methods used in the included literature are basically IHC. When it is used to detect the expression of ERCC1, there are differences in the scoring criteria among the studies. (2) The scope of the selected research is narrow, mostly in Asian countries, only 9 in Europe and America, but none in Africa and other countries. (3) Although IHC is used to detect the expression of ERCC1 in all of the studies, the manufacturer, dilution concentration, and judgment criteria of antibody are not completely consistent, which may affect the results of meta-analysis. (4) The test of publication bias on the overall study and the population in Asian countries shows that there is still a partial publication bias. However, these results should be interpreted with caution, as the criteria for judging gene expression are inconsistent in the selected studies. Sensitivity tests may also be necessary. In addition, prospective studies with larger sample sizes still need to further confirm these findings.

In conclusion, there is a correlation between the high expression of ERCC1 and the chemosensitivity of NSCLC platinum drugs. The low expression of ERCC1 is more sensitive to platinum chemotherapy in patients with advanced NSCLC, especially in Asian population, but not in Caucasians. The state of ERCC1 may be a potential biomarker for predicting the efficacy of platinum chemotherapy in NSCLC. However, considering the limitations of this study, large-scale and well-designed studies are still needed to investigate the factors that may affect the response to platinum chemotherapy.

## Figures and Tables

**Table 1:** Characters of included studies.

First author	Year	Country	Method	Stage	Therapy regimen	Chemotherapy course	ERCC1 positive		ERCC1 negative		NOS score
							Response	No response	Response	No response	
Lee	2009	Korea	IHC	IIIB-IV	Platinum-based	≥2	11	17	7	15	8
Ota	2009	Japan	IHC	IV	Platinum-based	≥2	26	74	15	41	8
Azuma	2009	Japan	IHC	IB-IIIB	Cisplatin-based	≥2	6	10	15	3	8
Wang	2009	China	IHC	IIIB-IV	Cisplatin-based	≥2	12	13	17	11	7
Shan	2009	China	IHC	IIIB-IV	Platinum-based	≥2	7	22	33	19	7
Wang	2009	China	IHC	III	Cisplatin-based	≥2	3	11	9	4	6
Wang	2010	China	IHC	IIIB-IV	Platinum-based	≥2	14	29	44	37	8
Li	2010	China	IHC	IIIB-IV	Cisplatin-based	≥2	3	30	11	27	7
Yang	2011	China	IHC	IIIB-IV	Cisplatin-based	≥2	4	16	13	14	7
Vassalou	2013	Greece	IHC	IIIB-IV	Platinum-based	≥2	13	42	14	25	8
Fumie	2013	Australia	IHC	III-IV	Platinum-based	≥2	5	23	8	15	8
Gao	2013	China	IHC	IIIA-IV	Platinum-based	≥2	21	54	19	53	7
Han	2017	Korea	IHC	IIIB-IV	Platinum-based	≥2	50	127	26	76	8
Honma	2018	Brasil	IHC	III-IV	Platinum-based	≥2	5	9	13	19	8

**Table 2:** Meta-analysis between ERCC1 expression and platinum chemosensitivity of NSCLC.

Items	Subgroup	*n*	OR	95% CI	*P*	*I* ^2^	*P* for heterogeneity	Analysis model	*P* for publication bias (Egger)	*P* for publication bias (Begg)
Overall		14	0.53	0.30∼0.79	<0.001	53.9	0.008	REM	0.014	0.063
Ethnicity	Asian	11	0.5	0.31∼0.82	0.001	63.7	0.002	REM	0.027	0.161
	Caucasian	3	0.56	0.30∼1.07	0.088	0.0	0.839	FEM	0.867	0.602
Tumor stage	III-IV	13	0.57	0.39∼0.84	0.005	50.0	0.02	REM	0.029	0.100
	Others	1	0.12	0.02∼0.59	0.009	NA	NA	NA	NA	NA

## Data Availability

The datasets used and/or analyzed during the present study are available from the corresponding author on reasonable request.
